# Can molar incisor hypomineralization cause dental fear and anxiety or influence the oral health-related quality of life in children and adolescents?—a systematic review

**DOI:** 10.1007/s40368-021-00631-4

**Published:** 2021-06-10

**Authors:** B. Jälevik, N. Sabel, A. Robertson

**Affiliations:** 1 Department of Pediatric Dentistry, Institute of Odontology at the Sahlgrenska Academy, University of Gothenburg, Gothenburg, Sweden; 2 Specialist Clinics for Pediatric Dentistry, Public Dental Service, VGR, Mölndal, Sweden

**Keywords:** Molar incisor hypomineralization (MIH), Dental fear and anxiety, Review

## Abstract

**Purpose:**

Molar Incisor Hypomineralization (MIH) are first molars with developmental enamel defects and are common findings in many child populations. The porous nature of MIH enamel and the presence of post-eruptive enamel breakdown leads to the presence of hypersensitivity and pain, which is often the patient’s main complaint and can result in dental fear and affect the quality of life.

The present review aims to summarise the evidence for the ability of MIH to cause problems, such as dental fear and anxiety (DFA) and to summarise the evidence for a possibly negative impact on the oral health-related quality of life (OHRQoL) of MIH affected children and adolescents, in a systematic review.

**Method:**

Two searches, (1) MIH AND dental anxiety and (2) MIH AND Quality of life, were performed in MEDLINE/PubMed and Scopus. Selection demands were fulfilling the MIH diagnosis criteria using validated instruments and questionnaires for assessing DFA and OHRQoL, respectively.

**Results:**

After removing duplicates and articles not fulfilling the selection demands, 6 studies concerning MIH and DFA and 8 studies concerning MIH and OHRQoL remained.

**Conclusion:**

Children and adolescents with diagnosed MIH did not seem to suffer from increased dental fear and anxiety, but indicated an impaired oral health-related quality of life.

## Introduction

First permanent molars (FPM), which at eruption show areas of hypomineralised enamel, were primarily observed in the 1980s (Koch et al. 1987, Suckling et al. 1987). For the past 20 years, the condition, which has been termed Molar-Incisor Hypomineralization (MIH) (Weerheijm et al. 2001), has attracted increasing attention and proved to occur all over the world. A comprehensive literature research, with 70 eligible prevalence studies included, found a pooled MIH prevalence of 14.2%, globally (Zhao et al. 2018).

Clinically, the porous hypomineralised areas appear as white–yellow to brownish, well-defined opaque spots. In these areas, the enamel is insufficiently mineralised with varying degrees and can fall apart, especially on the occlusal surfaces of the teeth (Weerheijm et al. 2003). The severity of MIH has a significant impact on the treatment need in the FPM. Already restored MIH molars remain within short re-treatment cycles. (Leppaniemi et al. 2001). It has been shown that children with MIH have undergone dental treatment of their FPMs approximately ten times as often as children without MIH (Jälevik and Klingberg 2012).

The porous nature of MIH enamel and the presence of post-eruptive enamel breakdown leads to hypersensitivity and pain, which is often the patient’s main complaint and can affect the quality of life. There is also the increased risk of dental fear and anxiety (Jälevik and Klingberg 2002).

Teeth with hypersensitivity not only create problems for patients, but also for the dentists. The sensitivity becomes problematic when it hinders the possibility of obtaining sufficient pain control with, consequently, behavioural management problems due to dental fear and anxiety, which is related to the pain experienced by the patients during multiple treatment appointments (Raposo et al. 2019; Jälevik and Klingberg 2012).

Today, approximately 500 scientific articles have been published concerning various aspects of MIH, including biochemical and histomorphological properties, diagnostics, prevalence, aetiology, treatment, dental fear and quality of life (D3 group). Approximately, 40 review articles have been published on various aspects of MIH (D3-group).

Conditions that mainly affect the oral health-related quality of life are untreated dental caries, dental trauma, increased dental protrusion, wearing orthodontic appliances and severe periodontal disease (De Stefani 2019). However, there are no reviews regarding the impact of MIH on dental fear, treatment problems and quality of life.

In the literature, it is often stated that MIH has created problems for both the affected children and treating dentists in the form of dental fear, treatment problems and that MIH has contributed to a poorer quality of life for the affected children (Jälevik and Klingberg 2002). It has been suggested that children, affected by oral and orofacial disorders, such as MIH, have compromised functioning, well being and quality of life (QoL) (Kalkani et al. 2016, Barbosa and Gavião 2008). The evidence for these proposals is regarded as weak since it is mainly based on the observations by the treating dentists and occasionally by the parents, yet seldom by the affected children.

The present research question is: Are children and adolescents with diagnosed MIH more anxious in the dental context and/or do the impaired teeth influence their oral health-related quality of life?

Systematic reviews are essential tools for summarising evidence accurately and reliably. This review aims to summarise the evidence for the ability of MIH to cause problems, such as dental fear and anxiety and to summarise the evidence for a possibly negative impact on the oral health-related quality of life (OHRQoL) of MIH affected children and adolescents, in a systematic review.

Assessment of behaviour management problems has been partly based on observations of the child's behavior during treatment and partly based on the dentist's notes in the dental records, clearly expressing delay of treatment or rendering treatment impossible. This type of registration is subjective and almost certainly biased; therefore, this review has refrained from including this item.

## Methods

This systematic review followed a protocol in accordance with the Preferred Reporting Items for Systematic Reviews and Meta-Analysis (PRISMA). Registered in PROSPERO: CRD42021233962.

### Searches

Searches were performed in MEDLINE/PubMed and Scopus. To find the eligible articles, a search strategy was performed combining MeSH terms, MeSH synonyms and free terms. The ‘AND’ and ‘OR’ Boolean operators were applied to combine keywords (Tables 1, 2). Searches performed from inception dates to date noted. Once selected, the references were analysed according to the eligibility criteria.

**Table 1:** Search 1 MIH AND dental anxiety

Search order	Search string	No results
PubMed 2020–11-03		
#13	#9 AND #12	34
#12	#10 OR #11	255,913
#11	Anxiety[tiab] OR anxieties[tiab] OR fear[tiab] OR odontophobia[tiab] OR phobia[tiab] OR phobias[tiab] OR DAQ[tiab] OR CFSS-DS[tiab]	255,158
#10	Dental anxiety[mesh]	2683
#9	#3 OR #8	8485
#8	#6 AND #7	5633
#7	Hypominerali*[tiab] OR mottling[tiab] OR opacit*[tiab] OR cheese[tiab] OR hypoplasia*[tiab] OR idiopathic enamel[tiab] OR opaque[tiab] OR calcification[tiab] OR discoloration[tiab]	121,128
#6	#4 OR #5	271,505
#5	Molar*[tiab] OR tooth[tiab] OR teeth[tiab]	245,897
#4	Molar[mesh] OR tooth[mesh]	89,132
#3	#1 OR #2	4393
#2	Dental enamel hypoplasia[tiab] OR hypoplastic Enamel[tiab] OR enamel agenesis[tiab] OR enamel ageneses[tiab] OR enamel hypoplasia[tiab] OR enamel hypoplasias[tiab] OR Molar Incisor Hypomineralization[tiab] OR enamel hypomineralization[tiab] OR MIH[tiab] OR molar Hypomineralization[tiab] OR molar Incisor Hypomineralisation[tiab] OR enamel hypomineralisation[tiab] OR Molar hypomineralisation[tiab] OR enamel defect[tiab] OR enamel defects[tiab]	2810
#1	Dental enamel hypoplasia[mesh]	2792
Scopus 2020–11-03		
#8	Limit to article, review	80
#7	#5 AND #6	91
#6	TITLE-ABS-KEY(anxiety OR anxieties OR fear OR odontophobia OR phobia* OR DAQ OR CFSS-DS)	536,099
#5	#1 OR #4	18,611
#4	#2 AND #3	16,203
#3	TITLE-ABS-KEY (molar* OR tooth OR teeth)	659,774
#2	TITLE-ABS-KEY(hypominerali* OR mottling OR opacit* OR cheese OR hypoplasia* OR “idiopathic enamel” OR opaque OR calcification OR discoloration)	289,257
#1	TITLE-ABS-KEY(“dental enamel hypoplasia” OR “hypoplastic enamel” OR “enamel agenesis” OR “enamel ageneses” OR “enamel hypoplasia*” OR “Molar Incisor Hypomineralization” OR “enamel hypomineralization” OR MIH OR “molar Hypomineralization” OR “Molar Incisor Hypomineralisation” OR “enamel hypomineralisation” OR “molar Hypomineralisation” OR “enamel defect*”)	5235

**Table 2:** Search 2 MIH AND Quality of life

Search order	Search string	No results
PubMed 2020–11-03		
#13	#9 AND #12	156
#12	#10 OR #11	540,874
#11	Life quality[tiab] OR life qualities[tiab] OR QoL[tiab] OR HRQoL[tiab] OR HRQL[tiab] OR OHRQoL[tiab] OR personal satisfaction[tiab] OR patient satisfaction[tiab] OR Activities of Daily Living[tiab] OR personal autonomy[tiab] OR patient preference[tiab] OR patient preferences[tiab] OR self-concept[tiab] OR CPQ11-14[tiab]	144,445
#10	Quality of Life[mesh] OR personal satisfaction[mesh] OR patient satisfaction[mesh] OR Activities of Daily Living[mesh] OR personal autonomy[mesh] OR self-concept[mesh]	484,730
#9	#3 OR #8	8485
#8	#6 AND #7	5633
#7	Hypominerali*[tiab] OR mottling[tiab] OR opacit*[tiab] OR cheese[tiab] OR hypoplasia*[tiab] OR idiopathic enamel[tiab] OR opaque[tiab] OR calcification[tiab] OR discoloration[tiab]	121,128
#6	#4 OR #5	271,505
#5	Molar*[tiab] OR tooth[tiab] OR teeth[tiab]	245,897
#4	Molar[mesh] OR tooth[mesh]	89,132
#3	#1 OR #2	4393
#2	Dental enamel hypoplasia[tiab] OR hypoplastic Enamel[tiab] OR enamel agenesis[tiab] OR enamel ageneses[tiab] OR enamel hypoplasia[tiab] OR enamel hypoplasias[tiab] OR Molar Incisor Hypomineralization[tiab] OR enamel hypomineralization[tiab] OR MIH[tiab] OR molar Hypomineralization[tiab] OR Molar Incisor Hypomineralisation[tiab] OR enamel hypomineralisation[tiab] OR molar hypomineralisation[tiab] OR enamel defect[tiab] OR enamel defects[tiab]	2810
#1	Dental enamel hypoplasia[mesh]	2792
Scopus 2020–11-03		
#8	Limit to article, review	378
#7	#5 AND #6	399
#6	TITLE-ABS-KEY ("Quality of Life" OR "life quality" OR "life qualities" OR "personal satisfaction" OR QoL OR HRQoL OR HRQL OR OHRQoL OR "patient satisfaction" OR "Activities of Daily Living" OR "personal autonomy" OR "patient preference” OR "self-concept” OR CPQ11-14)	869,714
#5	#1 OR #4	18,611
#4	#2 AND #3	16,203
#3	TITLE-ABS-KEY (molar* OR tooth OR teeth)	659,774
#2	TITLE-ABS-KEY(hypominerali* OR mottling OR opacit* OR cheese OR hypoplasia* OR “idiopathic enamel” OR opaque OR calcification OR discoloration)	289,257
#1	TITLE-ABS-KEY(“dental enamel hypoplasia” OR “hypoplastic enamel” OR “enamel agenesis” OR “enamel ageneses” OR “enamel hypoplasia*” OR “Molar Incisor Hypomineralization” OR “enamel hypomineralization” OR MIH OR “molar hypomineralization” OR “Molar Incisor Hypomineralisation” OR “enamel hypomineralisation” OR “molar hypomineralisation” OR “enamel defect*”)	5235

#### Selection process

**Fig. 1:**
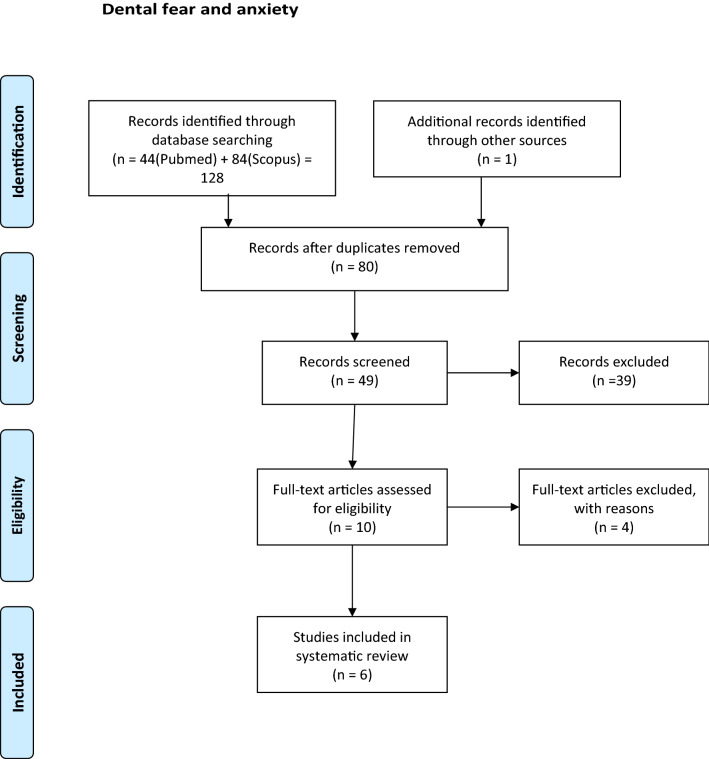
Flow fear. From: Moher D, Liberati A, Tetzlaff J, Altman DG, The PRISMA Group (2009). Preferred Reporting Items for Systematic Reviews and Meta-Analyses: The PRISMA Statement. PLoS Med 6(6): e1000097. doi:10.1371/journal.pmed1000097

**Fig. 2:**
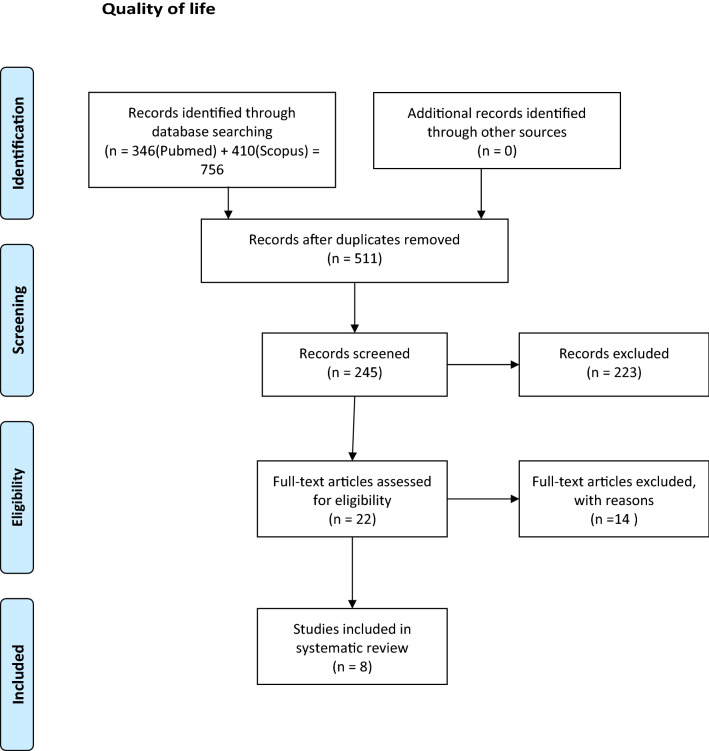
Flow chart quality of life. From: Moher D, Liberati A, Tetzlaff J, Altman DG, The PRISMA Group (2009). Preferred Reporting Items for Systematic Reviews and Meta-Analyses: The PRISMA Statement. PLoS Med 6(6): e1000097. doi:10.1371/journal.pmed1000097

Duplicates were removed and after the screening of abstracts, possible articles for inclusion were read by three authors (BJ, NS, AR) in full text. After reading, discussions and agreement, articles not fulfilling the inclusion criteria were removed (Figs. 1, 2).

### *Inclu*s*ion criteria*

- Full text articles published in peer reviewed journals
- MIH diagnosis in accordance with established MIH criteria, e.g., DDE index (FDI Working Group 1992) or EAPD index (Weerheijm et al. 2003)
- Dental fear and anxiety (DFA) and oral health-related quality of life (OHRQoL) registered in accordance with validated scales or questionnaires
- Written in the English language

The PICOT strategy applied for inclusion criteria:

- Population (P): 6-to 18-year-old children.
- Intervention (I): Not applicable.
- Comparator (C): MIH.
- Outcome (O): (a) Dental Fear and Anxiety (DFA), (b) Oral Health-Related Quality of Life (OHRQoL).
- Type of study (T): Case–Control and Cross-Sectional studies.

### Exclusion criteria

- MIH diagnosis not in accordance with established MIH indices
- DFA or OHRQoL not measured with validated scales or questionnaires
- Reviews, case reports, letters to editor

### Assessment of risk of bias in included studies

All three authors (BJ, NS, AR), independently, assessed the risk of bias for the eligible articles using a modified version of the quality assessment for prevalence studies tool developed by Munn et al. (2015).

### Data extraction

#### Population

- The country where the study was conducted.
- Type of study.
- Size and age of the study group.

### Criteria for MIH diagnosis

Number of children with MIH diagnosis and degree of disorder.

Method for reporting Dental Fear and Anxiety and/or Oral Health-Related Quality of Life.

Respondent: Child or parents.

## Results

### Data searches

After duplicates were removed, the number of articles identified in database searches was 80 for DFA (Dental Fear and Anxiety) and 511 for OHRQoL (Oral Health-Related Quality of Life). After screening the abstracts, 10 articles for DFA and 22 articles for OHRQoL remained for complete reading. The articles were read by two authors: DFA (AR and BJ) and OHRQoL (NS and BJ). After reading and discussions, 4 DFA articles and 14 OHRQoL articles were excluded. The most common reason for exclusion (10 articles) was that nonvalidated instruments/questionnaires were used. Four articles were excluded as it was not the effect of having MIH but the result after treatment of MIH that was measured and 4 because there was no valid MIH diagnosis (Tables 3, 4). Six articles remained regarding how MIH affected DFA (Tables 5, 6). Eight articles dealt with MIH's impact on OHRQoL (Table 7).

**Table 3:** Excluded papers: dental fear and anxiety

Authors	Articles	Motives
Crombie et al. (2008)	Molar incisor hypomineralization: a survey of members of the Australian and New Zealand Society of Paediatric Dentistry	No fear measure instrument
Kalkani et al. (2016)	Molar incisor hypomineralization: experience and perceived challenges among dentists specialising in paediatric dentistry and a group of general dental practitioners in the UK	No fear measure instrument
Mahoney (2012)	Molar incisor hypomineralization	No fear measure instrument
Rolim et al. (2021)	Adhesive restoration of molars affected by molar incisor hypomineralization: a randomised clinical trial	Treatment evaluation

**Table 4:** Excluded papers: quality of life

Authors	Article	Motives
Almuallem et al. (2018)	Molar incisor hypomineralization (MIH) – an overview	OHRQoL vs MIH not measured
Andrade et al. (2019)	Impact of developmental enamel defects on quality of life in 5‐year‐old children	5-year-olds, no MIH diagnosis
Ebel et al. (2018)	The severity and degree of hypomineralization in teeth and its influence on oral hygiene and caries prevalence in children	OHRQoL vs MIH not measured
Elhennawy et al. (2019)	Outcome and comparator choice in molar incisor hypomineralization (MIH) intervention studies: a systematic review and social network analysis	Review, OHRQoL vs MIH, not measured
Fütterer et al. (2020)	Influence of customised therapy for molar incisor hypomineralization on children's oral hygiene and quality of life	OHRQoL vs MIH not measured. Treatment evaluation
Hasmun et al. (2020)	Determinants of children’s oral health-related quality of life following T aesthetic treatment of enamel opacities	OHRQoL vs MIH not measured. Treatment evaluation
Hasmun et al. (2018)	Change in Oral Health-Related Quality of Life Following Minimally Invasive Aesthetic Treatment for Children with Molar Incisor Hypomineralization: A Prospective Study	OHRQoL vs MIH not measured. Treatment evaluation
Kalkani et al. (2016)	Molar incisor hypomineralization: experience and perceived challenges among dentists specialising in paediatric dentistry and a group of general dental practitioners in the UK	Evaluation of dentist perception
Large et al. (2020)	What children say and clinicians hear: accounts relating to incisor hypomineralization of cosmetic concern	Estetic evaluation
Marshman et al. (2009)	The impact of developmental defects of enamel on young people in the UK	No MIH diagnosis. Qualitative study, no questionnaire
Oyedele et al. (2015)	Co-morbidities associated with molar-incisor hypomineralization in 8 to 16 year old pupils in Ile-Ife, Nigeria	No validated QoL questionnaire
Paglia (2018)	Molar Incisor Hypomineralization: paediatricians should be involved as well!	Letter to editor
Sujak et al. (2004)	Esthetic perception and psychosocial impact of developmental enamel defects among Malaysian adolescents	No validated QoL questionnaire, no MIH diagnosis
Vargas-Ferreira and Ardenghi (2011)	Developmental enamel defects and their impact on child oral health-related quality of life	No MIH diagnosis

**Table 5:** Dental fear and anxiety description

Authors	Country	Study design	Sample size	Age years	Boys *N*	Girls *N*	MIH diagnos	Severe MIH *N*	Mild MIH *N*	MIH (severe/mild) %
Arrow (2017)	Australia	Cross sectional	88	15			DDE index	18*		20
Laureno et al. (2020)	Brazil	Cross sectional	466	8–10			Ghamin 2015	19	37	12 (4/8)
Jälevik et al. (2002)	Sweden	Case control	73	9	35	38	DDE index	32		
Jälevik et al. (2012)	Sweden	Case control	67	18	31	36	DDE index	30		
Kosma et al. 2016	Greece	Cross sectional	1179	8	1139	1196	EAPD 2003	33	221	22 (3/19)
			1156	14				85	159	21 (7/14)
Menoncin et al. (2019)	Brazil	Cross sectional	731	8	357	374	EAPD 2003	88*		12

*p < 0.05

**Table 6:** Dental fear and anxiety, findings

Authors	CFSS-DS mean (SD)			DAQ reported DA			DVSS mean			MCDAS mean (SD)			Respondent
	No MIH	MIH		no MIH *N* (%)	MIH *N* (%)		No MIH	MIH		No MIH	MIH		
Arrow (2017)										21.0 (8.2)	18.9 (7.1)	NS	Parent
Carvalho Laureno et al. (2020)	29.87 (0.92)		NS										Child
Jälevik et al. (2002)	20.8 (5.4)	23.3 (7.5)	NS	8 (20)	14 (44)	*							Parent
Jälevik et al. (2012)	21.7 (5.8)	22.0 (6.2)	NS				39.6	38.2	NS				Child
Kosma et al. 2016	25.3 (10.5)	25.0 (9.5)	NS										Child
	27.2 (9.2)	28.1 (9.2)	NS										
Menoncin et al. (2019)				348 (48)	57 (64)	NS							Parent

*CFSS-DS *Dental Subscale of Children’s Fear Survey Schedule; *DAQ *Dental Anxiety Question; *DVSS *Dental Visit Satisfaction Scale; *MCDAS *Modified Child Dental Anxiety Scale; *NS *No significance

*p < 0.05

**Table 7:** QoL studies

Authors	Country	Study design	Sample size *N*	Age years	Boys *N*	Girls *N*	MIH diagnos	Severe MIH *N*	Mild MIH *N*	Presence of MIH (severe/mild) %	COHQoL	Responder	Summary of finding
Arrow (2013)	Austr	Cross Sectional	550	7	?	?	DDE index	32	86	21% (6%/16%)	P-CPQ	P	No impact of MIH on OHRQoL is shown
Arrow (2017)	Austr	Cross Sectional	88	14–16	?	?	DDE index	18		20%	CPQ_11-14_	C	No impact of MIH on OHRQoL is shown
Dantas-Neta et al. (2016)	Brazil	Cross Sectional	594	11–14	?	?	EAPD 2003	25	84	18% (4%/14%)	P-CPQ	P	Severe MIH was significantly associated with a greater negative impact of the ‘functional limitation domain’ according to parents’/caregivers’ perceptions. According to the children, severe MIH was significantly associated with a greater negative impact of the ‘oral symptom domain’ and ‘functional limitation domain’
											CPQ_11-14_	C	
Dias et al. (2020)	Brazil	Cross Sectional	42	6–7	125	128	EAPD 2003	15	238		CPQ_8-10_	C	Those with severe MIH are compared to those with mild. The results of OHRQoL questionnaire indicate a certain but not significant impact on the domains: ‘Oral symptoms’, ‘Functional limitations’ and also ‘Emotional wellbeing’ (the parents)
			211	8–12							CPQ_11-14_	C	
											P-CPQ	P	
Folayan et al. (2018)	Nigeria	Cross Sectional	428	6–9	438	415	EAPD 2003	20		3%	Child OIPD	C	No impact of MIH on OHRQoL is shown
			425	10–16				5					
Gutiérrez et al. (2019)	Mexico	Cross Sectional	411	8–10	194	217	EAPD 2003	166		40%	CPQ_8-10_	C	MIH, severe as well as mild, impact on OHRQoL in all domains (*p* < 0.001)
Portella et al. (2019)	Brazil	Cross Sectional	728	8–10	372	356	EAPD 2003	25	63	12% (3%/9%)	CPQ_8-10_	C	MIH, severe as well as mild, impact on OHRQoL the domain ‘oral symptoms’ (*p* < 0.001)
Velandia et al. (2018)	Colomb	Case Control	88	8–10	41	47	EAPD 2003	44			CPQ_8-10_	C	MIH, severe as well as mild, impact on OHRQoL in all domains (*p* < 0.001)

Description and findings

*C *Children, *P *Parent, *? * not given

### Populations

Most studies are of the cross-sectional type. Three studies are case–control studies (Jälevik and Klingberg 2002, 2012; Velandia et al. 2018).

The study groups varied in age and size. The age range in the study groups within the OHRQoL articles varied between 6 and 16 years to only one age. The most common age group was 8–10 years.

Within the DFA articles, all but one study included only one age group. In one DFA article, the study group was 8–10 years old (Table 5).

Latin America dominates as a country of origin for studies on OHRQoL (Table 7). Regarding DFA, studies have been conducted in Europe, Brazil and Australia (Table 5).

### MIH diagnosis

Regarding MIH diagnosis, most studies use the EAPD index (Weerheijm 2003). Four articles (Jälevik and Klingberg 2002, 2012, Arrow 2013, 2017) use the DDE index (FDI Commision on Oral Health 1992).

Five out of 14 articles did not report if the MIH disorder was mild or severe. Instruments and Questionnaires.

#### DFA

In 4 out of 6 DFA studies, CFSS-DS (Dental Subscale of Children’s Fear Survey Schedule) was used as a measuring instrument. Two of these studies were supplemented with other instruments, DAQ (Dental Anxiety Question) (Neverlien 1990) and DVSS (Dental Visit Satisfaction Scale) (Corah et al. 1988), respectively.

Another study used DAQ and one used MCDAS (Modified Child Dental Anxiety Scale) (Howard and Freeman 2007).

The *CFSS-DS* is the most frequently used measure of DFA in children and adolescents (Klingberg and Broberg 2007) and comprises 15 items scored on a Likert-type scale, ranging from 1 (not afraid at all) to 5 (very afraid). The total score ranges between 15 and 75.

*DAQ* consists of one question: “Do you think that your child is afraid of going to the dentist?” with four possible answers: no = 1; a little = 2; yes, he/she is afraid = 3; and yes, he/she is very afraid = 4.

*DVSS* has been developed to measure different aspects of the dentist–patient relationship from the patient´s point of view.

Modified Child Dental Anxiety Scale (MCDAS) was utilised in one study (Tables 5, 6).

#### OHRQoL

To evaluate MIH's impact on the affected children's quality of life, CPQ_8-10_ and CPQ_11–14_ (Jokovic et al. 2002) were mainly used. In two of these studies, a questionnaire for parents (P-CPQ) (Jokovic et al. 2003) was added and in one study, only the parental questionnaire was used. One study used Child-OIPD (Gherunpong et al. 2004).

Oral Health-Related Quality of Life focuses on oral health and orofacial concerns. It describes the way in which oral health affects a person’s ability to function, psychological status, social factors and pain or discomfort. Therefore, the OHRQoL attempts to represent the subjective side of oral health (Bekes and Hirsch 2013).

CPQ_8-10_, CPQ_11–14_ and P-CPQ are questionnaires within the entity OHRQoL, which was developed to suit different groups of responders. It contains questions in four domains: Oral symptoms, functional limitations, emotional well-being and social well-being.

Child-OIPD is another questionnaire within the OHRQoL entity developed by Gherunpong et al. (2004) (Table 7).

### Respondents

The parents responded in 3 out of 6 articles regarding DFA.

Concerning OHRQoL, the parent was the only respondent in one article; in two articles, parents and the child responded to one adjusted questionnaire each. In the remaining articles, the child was the respondent.

### Outcomes

#### DFA

A significant connection between MIH and DFA is only shown by DAQ in Jälevik & Klingberg (2002). The same study also showed a significant association of MIH and DFA with CFSS-DS, when using a cutoff value for DFA. CFSS-DS as well as DAQ were answered by the parents. Other studies only showed a tendency for increased DFA in MIH, but without significance (Table 6).

#### OHRQoL

Two of the articles showed a significant impact of MIH on all domains of OHRQoL (Gutiérrez et al. 2019; Velandia et al. 2018). Portella et al. (2019) found a significant impact on the domain "oral symptoms". Two studies (Dantas-Neta et al. 2016; Dias et al. 2020) demonstrated some influence in the domains Oral Symptoms and Functional Limitations. In the latter study, those with severe MIH were compared with those with severe MIH. In all other studies, those with MIH were compared with those without MIH regarding DFA as well as OHRQoL.

The results in the studies where both the parents and the children were respondents, in their respective questionnaires, indicated good consensus (Table 7).

## Discussion

In the present literature research, few articles were found that describe the ability of the condition Molar Incisor Hypomineralization (MIH) to cause dental fear and anxiety (DFA) and to impact on the quality of life (QoL) negatively in the affected children.

Comparison between the selected studies should be interpreted with caution due to the lack of uniformity in sample size and selection, diagnostics and age groups. Furthermore, the demographic and socioeconomic aspects, as well as the organisation of dental service for children, differ substantially around the world.

Most studies used the EAPD index when diagnosing MIH. A few older studies used the DDE index. As the EAPD index is based entirely on the DDE index, it is unproblematic.

### DFA

Most studies in the present review used the CFSS-DS instrument when searching for DFA among children with MIH. When comparing mean values between the groups (MIH vs no MIH), none of these studies found a significant difference. One study showed a significant difference when calculating a cutoff value for DFA, defined by total sample mean score plus one standard deviation, where the parents filled in the questionnaires (Jälevik and Klingberg 2002).

However, more recent studies have shown that a lot of surrounding factors can influence the CFSS-DS value. The parent as the informant has been questioned; the child alone as the informant has been advocated (Klingberg and Broberg 2007, Gustafson et al. 2010). Also, the calculation of the cutoff value for CFSS-DS is now being challenged. It should be adapted to age and gender (Lopes et al. 2013).

Moreover, a more recent review study (Cianetti et al. 2017) has shown that in studies using CFSS-DS ratings, the prevalence and the mean score of dental fear/anxiety was lower in Northern Europe than the remaining countries, the prevalence decreased with increasing age and the frequency was higher in females than in males. It can be questioned whether the measuring instrument CFSS-DS is reliable when it comes to studying specified dental disorders influencing dental fear.

Of the 15 questions, only a small number of questions can be linked to poor experience with MIH teeth. This is especially true in those parts of the world where dental health is good and where children's contact with dental care is established at a young age.

According to Klingberg et al. (1995), only approximately one-fourth of the children with BMP suffered dental fear. Consequently, the perceived difficulties in treating MIH teeth mainly does not reflect fear of dental care, but problems due to sensitivity of the teeth and the problem of obtaining adequate anesthesia (Rodd et al. 2007).

### QoL

Oral Health-Related Quality of Life (OHRQoL) focuses on oral health and orofacial concerns. It describes the way in which oral health affects a person’s ability to function, psychological status, social factors and pain or discomfort. Therefore, the OHRQoL attempts to represent the subjective side of oral health (Bekes and Hirsch 2013).

The articles in the present review concerning the effect of MIH on OHRQoL are all recent and reflect a relatively new, but rapidly growing concept in dentistry. It is an aspect of dental health, addressing the patient's perception of whether his/her current oral health status has an impact upon his/her actual quality of life.

The results indicate that the MIH condition can impact OHRQoL, in particularly the domains ‘Oral symptoms’ and ‘Functional limitations’.

It is a common perception that treatment of children with MIH can be problematic (Garg et al. 2012; Gamboa et al. 2018). Teeth with decay are quickly attacked by caries, difficult to anesthetise and restore and children have difficulties in cooperating. The impact in daily life is of great concern when it comes to the holistic perspective of MIH.

In a recent questionnaire about perception and clinical management of molar incisor hypomineralization among dentists, the most cited barrier to care was the child’s behaviour, followed by difficulty in achieving local anesthesia (Wall and Leith 2020).

In a similar questionnaire, dentists reported increased sensitivity of the affected teeth as the most frequently encountered problem. They also recognise the negative effect this condition has on the quality of life of the affected children and their families (Kalkani et al. 2016).

It has been shown that patients benefit from desensitising therapy for MIH-damaged teeth and that treatment can reduce hypersensitivity and improve the ability to ensure oral hygiene, but also has a positive impact on the quality of life (Fütterer et al. 2020). These results underline the importance of treating MIH-damaged teeth to help relieve children's suffering and improve their quality of life (Raposo et al 2019).

## Conclusion

Children and adolescents with diagnosed MIH did not seem to suffer from increased dental fear and anxiety, but indicated an impaired oral health-related quality of life.

Most previous studies on the treatment of MIH are the views of the dentists, with just a few from the child's perspective. To help dentists manage optimal treatment of MIH teeth, more studies from different countries regarding the impact of MIH on OHRQoL is desirable.
